# IL36 indicating good prognosis in human Hepatocellular Carcinoma

**DOI:** 10.7150/jca.47106

**Published:** 2020-08-27

**Authors:** Miao Hu, Ying Tong, Haisheng Fang, Jie Tang, Lang Liu, Yanni Hu, Jingze Li, Lan Zhong

**Affiliations:** 1 Department of Gastroenterology, Shanghai East Hospital, Tongji University School of Medicine, Shanghai, China, No.150 Jimo Road, Pudong New Area, Shanghai, China.; 2 Clinical Laboratory, Cancer Hospital of Fudan University, Fudan University, Shanghai, China, No 270 Dong 'an road, Shanghai, China.; 3 Department of pathology, First Affiliated Hospital of Nanjing Medical University, Nanjing, China, No 300 Guangzhou road, Nanjing, China.; 4 Center of Digestive Endoscopy, Shanghai East Hospital, Tongji University School of Medicine, Shanghai, China, No.150 Jimo Road, Pudong New Area, Shanghai, China.

**Keywords:** Hepatocellular carcinoma (HCC), biomarker, interleukin 36 (IL36), prognosis

## Abstract

**Background and Aim:** Hepatocellular carcinoma (HCC) is the leading cause of cancer death in men before the age of 60 years in China. Interleukin (IL)36 played important roles in antitumor immune responses, but its role in HCC is still unknown. We aimed to explore the correlation between IL36 and prognosis of HCC patients.

**Methods:** The expression of IL36 was measured by Immunohistochemistry (IHC), serum Enzyme-linked Immunosorbent Assay (ELISA) and flow cytometry (FCM). Chi-square test was performed to analyze the relationship between IL36 expression and clinical parameters of HCC patients. The correlation between IL36 expression and prognosis of HCC patients was evaluated by Kaplan-Meier method and Cox regression analysis.

**Results:** The IL36 expression in HCC tumor samples was lower than that in paired peri-tumor samples; the analyses suggested that there was no correlation between IL36 expression and age, gender, and tumor size, but tight relationship between IL36 expression and liver cirrhosis, metastasis and some other clinical parameters. The results of Kaplan-Meier analysis indicated positive expression of IL36 could induce high survival rate of patients. The detection of IL36 with ELISA suggested that expression of IL36 in serum was the highest in patients of HCC, other than the chronic hepatitis patients and the healthy. The result of FCM suggested the expression of IL36 was higher in CD4+ T cells than other immune cells.

**Conclusions:** There is a close relationship between the expression of IL36 and the prognosis of HCC, higher expression of IL36 suggested better prognosis and longer survival of HCC.

## Introduction

Based on the data of International Agency for Research on Cancer, more than 750,000 new liver cancer cases and 695,900 cancer deaths occurred all over the world every year 1. It is the second largest cause of liver-related deaths in the Asia-Pacific region, which accounted for 43.6% of all liver-related deaths in the region 2. In China, HCC is the most commonly diagnosed cancer and the leading cause of cancer death in men before the age of 60 years. According to the WHO's 2015 Global Health Estimates (GHE) dataset, chronic hepatitis B infection accounted for the most part of all deaths due to liver cancer (53%), whereas hepatitis C accounted for 6% of such deaths 2, so it is universally accepted that HBV infection is the prodromal stage of most HCC in China. Generally, HCC has a poor prognosis with relatively low 5-year survival rates (approximately 10%) 3. In HCC, frequent tumor metastasis and high relapse rates are the major causes of high mortality, and many patients with HCC have not been diagnosed until the disease is in its advanced stages and is no longer treatable. This status is ascribed to 3 key factors: the lack of early diagnosis; the deteriorated condition of the cirrhotic liver from which most HCC cases develop; and the high resistance of HCC to chemotherapy 4. The most commonly used biomarker in HCC is alpa-feta protein (AFP), but the early diagnosis of HCC still remains a crucial issue currently due to the low sensibility or specificity limitation of AFP 5-7. As a result, it is still desperately needed to find more sensitive biomarker for HCC early detection, and early predicting the prognosis of HCC.

Interleukin (IL)36 is a group of cytokines in the IL1 family with inflammatory effect. It includes three separate molecules, IL36α, IL36β, and IL36γ, also known as IL-1F6, IL-1F8, and IL-1F9, respectively 8. These cytokines share the same receptor, IL36R (also known as IL-1Rrp2 or IL-1RL2) and IL-1RAcP 9. IL36 has been demonstrated as a pivotal mediator of skin inflammation and could induce the expression of some pro-inflammatory cytokines such as IL12, IL23, IL22, IL-36R signaling is also essential for the development of psoriasiform dermatitis in response to environmental cues such as Imiquimod through the pathogenic IL-23/IL-17/IL-22 axis 10. Recent data suggests that IL-36 can promote CD4+ T cell-mediated type 1 immune responses, and IL-36R signaling is involved in Th1 immune responses against mycobacterium bovis BCG *in vivo* 11. Furthermore, it was recently reported that IL36 played important roles in promoting antitumor immune responses 12, 13 and could be a promising candidate for assessing the prognosis of antitumor treatment 9; however, its role in predicting the prognosis of human HCC is unknown.

In our study, we aimed to detect the expression of IL36 in different patients, and investigate its association with the prognosis and clinical features of HCC.

## Methods and Materials

### Patients and specimens

A total of 150 HCC patients, 14 HBV chronic hepatitis patients and 20 healthy persons for controls were all treated in Shanghai East Hospital from 2009 to 2013. Surgical specimens of cancerous tissue, paired adjacent peri-tumor tissue were obtained from all HCC cases, in accordance with the Ethics Committee of Shanghai East Hospital after obtaining an informed consent. Patients' data on gender, age, tumor size, presence of metastasis, with or without liver cirrhosis/HBV infection, TNM stage, Child-Pugh stage, serum AFP level were collected. The sample included 64 men and 86 women, with a mean age of 46 years, range 37-74 years (median 59 years, mean 58.2 years in women; median 49.5 years, mean 49.5 years in men). None of these patients experienced any preoperative anti-tumor therapy. The detailed clinic pathological characteristics are summarized in Table 2. All tissue samples were incubated in RNA Later (Ambion, Austin, Texas, USA) at 4°C for 24 hours and stored at -80°C until RNA extraction.

### Immunohistochemistry (IHC)

In brief, formalin-fixed, paraffin-embedded tissue sections were dewaxed and the antigens retrieved. The primary antibody against IL36 (Proteintech, 21255-AP, USA) was diluted 1:40. Secondary anti-mouse antibodies were applied on slides for 15min at room temperature followed by diaminobenzidine and hematoxylin treatment. PBS was used in the same manner as a substitute for the primary antibody and used as negative control. Photographs of two representative fields were captured under high-power magnification (× 400), and identical settings were used for each photograph. The representative fields were defined as follows: If there were both high-expression and low-expression areas at × 100 magnification; then an image was captured at × 400 magnification which contained both a high-expression and low-expression area. Image-Pro Plus v6.0 software was used to count and measure integrated optical density (IOD), and mean IOD was calculated from two photographs per patient. X-tile plots were created for assessment of IL-36 expression, and optimization of cut-off points were based on the outcome of IOD value 14. Statistical significance was assessed using the cut-off score derived from 150 cases by a standard log-rank method, with *p* values obtained from a lookup table.

### Enzyme-linked immunosorbent assay (ELISA)

Blood sampling (3 ml) was carried out through vein puncture in 5AM to 6AM. After the serum was separated by centrifugation (1000 g, 5 min), all samples were kept in -80°C refrigerator until the time of assay. The concentrations of IL36 in serum was evaluated using ELISA by human Interleukin 36 Gamma/IL1F9 (IL36G) Kit (Abbexa, abx253593, UK).

### Flow cytometry (FCM)

Human peripheral blood mononuclear cells (PBMCs) were isolated from blood of healthy controls, chronic hepatitis B and HCC patients by Ficoll-Paque Plus (Sigma-Aldrich, USA) density-gradient centrifugation. For surface staining, PBMCs were labelled with the indicated human antibodies (CD4-PerCP-Cy5.5, 560650, BD bioscience USA) (CD8- APC-Cy7,557834, BD bioscience, USA) and human IL36 antibody (IL36-APC, NBP2-71834, Novus, Switzerland) in FACS buffer (2.5 ml PBS, 50 ml newborn calf serum and 0.5 g NaN3) for 30 min at 4°C avoiding light, then The samples were washed with cold FACS buffer and detected by flow cytometry (FCM) (Beckman, CytoFLEX, USA).

Follow-up: all the patients were prospectively followed after surgery according to a formulated schedule (refer to Barcelona-Clinic Liver Cancer Group staging (BCLC staging). Follow-up schedule was once every 3 months for the first 2 years, then once every 6 months for the next 3 years, and finally once a year. The follow-up time ranged from 8 months to 61 months (median, 27.3 months). At reference date (May 30, 2018), surviving patients were censored at their last consultation, and non-surviving patients were censored at their death date. Overall survival time was calculated from the date of the initial surgical operation to death.

### Statistical analysis

Statistical calculations were performed using SPSS18.0 software (SPSS Inc, USA). Differences between groups were estimated using Student's t test. Chi-squared test, Kaplan-Meier and Cox regression analysis were conducted to evaluate the correlations between groups and subgroups. Differences were considered significant if *P*<0.05.

## Results

**Figure 1:**
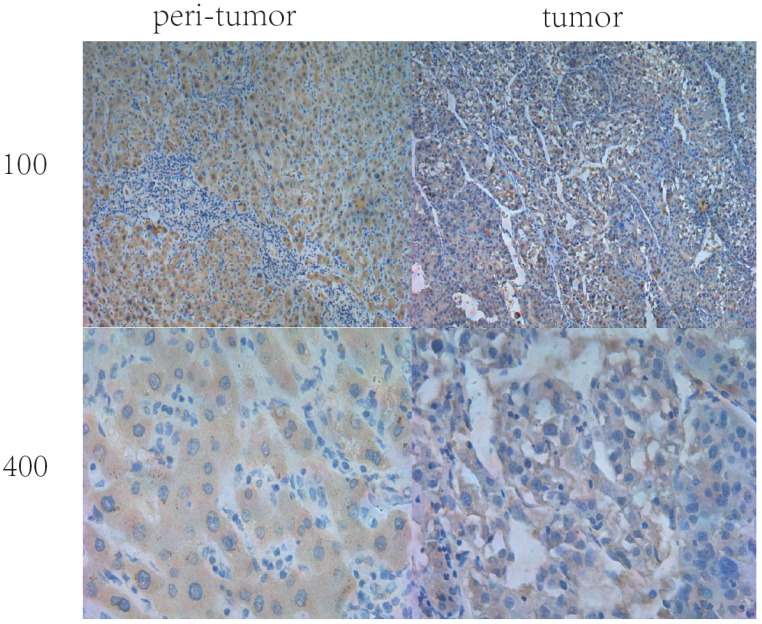
The IHC staining of IL36 in human HCC tumor tissues and peri-tumor tissues

Expression of IL36 in HCC tissues and paired peri-tumor (normal) tissues positive staining of IL36 displayed mainly in the cytoplasm (**Figure 1**) by immunohistochemical staining. In the tumor tissues, IL36 expression was positive in only 42 (28%) of 150 HCC specimens while in paired adjacent peri-tumor tissues, 132 cases (85%) were positive (**Figure 1, Table 1**). This suggested that the expression of IL36 in HCC was significantly lower in tumor tissues than that in the adjacent peri-tumor (normal) tissues (*P*<0.05).

### Correlation of IL36 expression with prognosis of HCC patients

**Figure 2:**
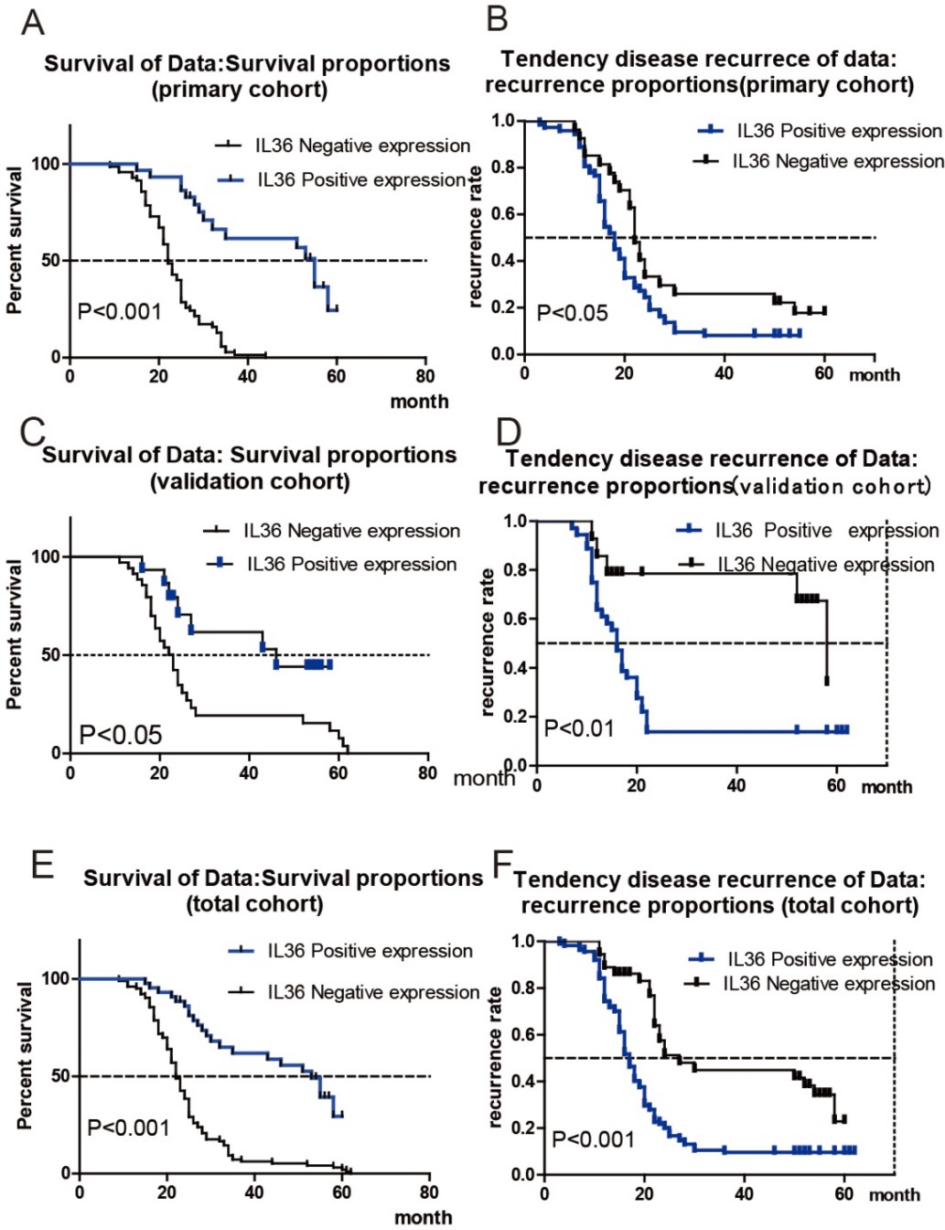
** The Correlation of IL36 expression with prognosis of HCC patients.** a the correlation between IL36 expression and survival of HCC patients in primary cohort. b the correlation between IL36 expression and recurrence of HCC patients in primary cohort. c the correlation between IL36 expression and survival of HCC patients in validation cohort. d the correlation between IL36 expression and recurrence of HCC patients in validation cohort. e the correlation between IL36 expression and survival of HCC patients in total cohort (primary cohort + validation cohort). f the correlation between IL36 expression and recurrence of HCC patients in total cohort (primary cohort + validation cohort).

Patients were divided into two cohorts randomly: the primary cohort (n=100) and the validation cohort (n=50). The correlation between IL36 expression and survival or recurrence of HCC patients was analyzed with Kaplan-Meier analysis. As shown in **Figure 2A**, HCC patients in the primary cohort with negative IL-36 expression had much shorter OS times, (mean OS 22 months vs 55 months, HR: 4.879, 95% CI: 3.03-7.86), while positive expression of IL-36 in HCC tissues had a lower tendency of disease recurrence (mean TTR 18 months vs 22 months; HR: 1.66, 95%CI: 1.05-2.63) (**Figure 2B**). Likewise, **Figure 2C** showed HCC patients in validation cohort with negative expression of IL36 had much shorter OS times (mean OS 23 months vs. 46 months, HR=2.43,95%CI (1.19-4.97)), and Figure 2D suggested a higher tendency of disease recurrence (mean TTR 16 months vs 58 months, HR=2.81,95%CI (1.37-5.74)) in HCC patients with negative IL36 expression in validation cohort. The association between IL-36 expression and OS/TTR was also analyzed in all 150 HCC patients, including both the primary cohort and validation cohort. The results showed that patients with negative IL-36 expression in HCC tissues had much shorter OS times (mean OS 22 months vs 53 months, HR: 3.56, 95% CI: 2.42-5.24 **Figure 2E**) and a higher tendency of disease recurrence (mean TTR 17 months vs. 27 months, HR: 2.33, 95%CI: 1.58-3.44, **Figure 2F**).

### Correlation between IL-36 expression and clinical pathological features

**Figure 3:**
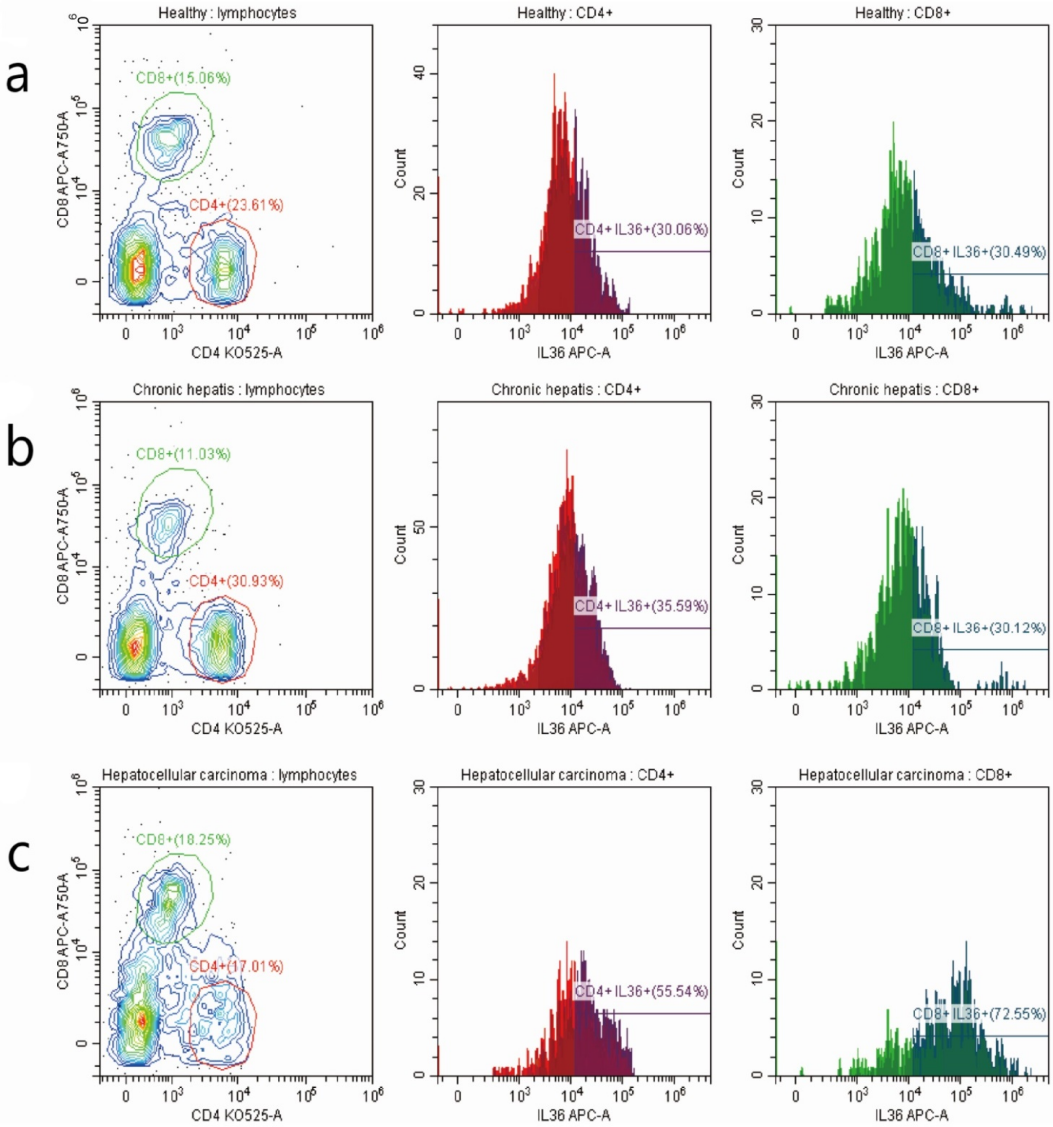
** The detection of T lymphocytes with FCM in PBMC of 3 groups of patients with different stages of hepatitis B infection.** a the detection of T cells in PBMC of healthy controls. b the detection of T cells in PBMC of hepatitis B patients. c the detection of T cells in PBMC of HCC patients. The number of patients in different groups by the detection of FCM (healthy controls (10 cases), chronic hepatitis B patients (10 cases), and HCC patients (8 cases)).

**Figure 4:**
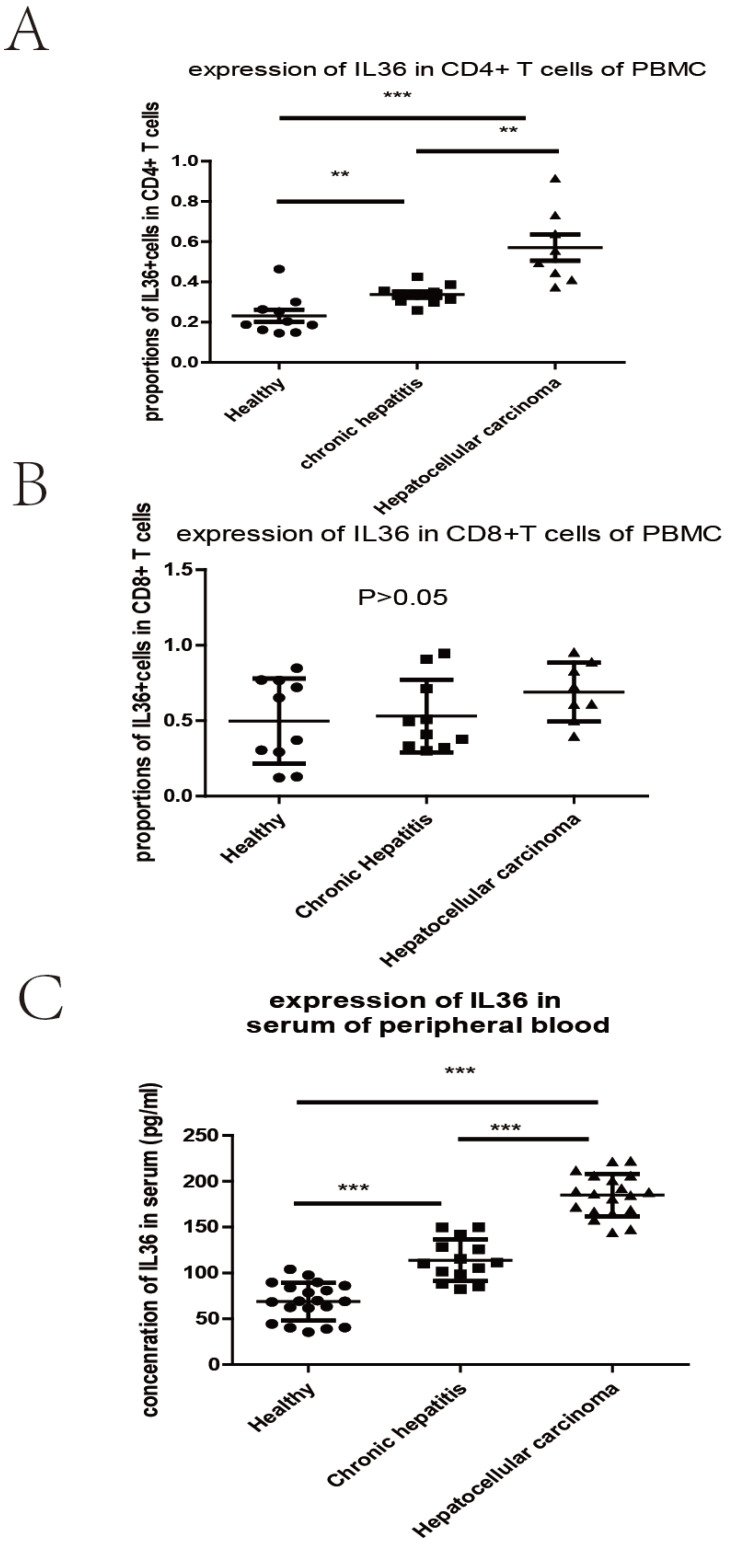
** The different expression of IL36 in 3 groups of patients detected by FCM and ELISA.** a the expression of IL36 in CD4 positive T cells of PBMC by FCM. b the expression of IL36 in CD8 positive T cells of PBMC by FCM. c the expression of IL36 in serum of peripheral blood by ELISA. The number of patients in different groups by the detection of ELISA (healthy controls (20 cases), chronic hepatitis B patients (14 cases), and HCC patients (19 cases)).

We investigated the relationship between the expression of IL-36 and the clinical parameters of HCC patients. Chi-square results showed that there were significant relationships between the expression of IL-36 and certain clinical parameters, such as liver cirrhosis, metastasis, HBV infection, serum AFP level, and surgical intervention (*P*<0.05); but no tight correlation was found between expression level of IL36 and other clinical parameters, likely age, gender, TNM stage and tumor size (*P*>0.05) (**Table 2**).The association between clinical pathological features and prognosis were further analyzed by univariate and multivariate analyses. By using multivariate analysis, it showed features such as age (younger than 55 years), positive IL36 staining and lower serum AFP level (≤400 ng/ml), predicted good prognostic capacity; with the univariate analysis, it suggested factors such as male, older than 55 years, with liver cirrhosis, Stage B (according to Child-Pugh classification) and palliative resection predicted poor prognosis (**Table 3**). IL36 expression in different subgroups of T lymphocyte in PBMC with FCM (**Figure 3 & Figure 4A, B**) Since IL36 may affect the immune systems 9, 15 we detected the expression of IL36 on T lymphocyte and its subtypes in different population (healthy controls, chronic hepatitis B patients, and HCC patients) by FCM. The results of FCM suggested that expression of IL36 was the highest in CD4^+^ T cells than other cells (**Figure 3 & Figure 4A, B**). Serum concentration of IL36 with ELISA (**Figure 4C**) results indicated that the serum concentration of IL36 was the highest in HCC patients group, the second was in the chronic hepatitis B patients group, and the lowest was in the healthy control group (**Figure 4C**).

## Discussion

HCC is an aggressive malignancy with an increasing rate of incidence and a poor prognosis 16, China has the highest incidence of liver cancer in the world 2, and there are 466,000 cases of liver cancer in China every year according to the China Cancer Research Report recently 17. Chronic hepatitis B is the main cause of HCC in China 2. Although surgery and transcatheter arterial chemoembolization (TACE) therapy for HCC have made enormous achievement recently; however, it is very common to meet a perishing prognosis for HCC patients after surgical resection 18. Currently, the most commonly used marker for HCC is serum α-fetal protein (AFP); however, approximately 50% of HCC patients are negative for this marker 19. Therefore, it is urgent to find a biomarker that can be used for early diagnosis, early screening and early prediction of prognosis.

The interleukin family is generally distributed in the tumor microenvironment, and many kinds of interleukin molecules play different roles in tumor progression and metastasis. As a newly discovered interleukin family member, IL-36 has been reported to play important roles in autoimmune disease 20, it was reported that the role of IL-36 has been demonstrated extensively in the skin, where it can act on keratinocytes and immune cells to induce a robust inflammatory response and is implicated strongly through functional and genetic evidence in the pathology of psoriatic disorders 20; and IL-36 family members play a key role in the differentiation and function of polarized innate and adaptive lymphoid cells 15. For Psoriasis progression, IL-36γ is the key ingredient in the dermal vascular compartment and is likely to enhance psoriatic skin inflammation by activating endothelial cells and promoting leukocyte recruitment 21; there were also several research pointing toward a critical function of IL-36 in the priming of Th1 cell responses *in vitro*, and in adaptive immunity in a model of mycobacterial infection *in vivo* 22; some other research suggested a beneficial effect of inhibition from IL-36γ/IL-17C axis-against anti-TNF-induced psoriasis form lesions in patients with inflammatory bowel disease 23. Anyway, IL36 are important activators of the inflammatory response by stimulating both innate and adaptive immune response. Furthermore, recent research demonstrates that IL36 plays critical roles in tumor treatment and prognosis. For example, IL36 gene therapy has therapeutic effects on the regression of tumor masses in fibrosarcoma mouse model 24, IL-36α could be applied as a novel predictor of prognosis and a potential therapeutic drug for colorectal cancer 25, and IL-36γ could play important role in the physiologic immune response to colorectal cancer by sustaining inflammation within the tumor microenvironment 26. However, the role of IL36 in detection and prognosis of HCC still remains unclear. Our results demonstrated IL-36 was significantly expressed in HCC tumor tissues compared with adjacent peri-tumor tissues. Those with positive IL-36 expression in this setting of patients had much longer OS times and lower tendency of cancer recurrence. Further investigation suggested a significant correlation between the expression level of IL-36 and combination with certain clinical facts, such as metastasis, AFP, HBV infection, and liver cirrhosis, but no tight association was established between the level of IL-36 and other clinical parameters, such as age, gender, tumor size and TNM classification. In the meantime, the correlation between clinical parameters and prognosis of patients with HCC was identified. Clinical parameters such as older than 55 years, negative IL36 expression, and AFP ≥400 µg/L predicted poor prognosis; IL36 level were the highest one in HCC patients compared with healthy and hepatitis B patients, which suggested that IL36 could be a novel biomarker for detection of HCC. Further, the data from FCM detection suggested expression of IL36 was higher in CD4^+^ T cells than in CD8^+^ T cells, and was the highest in HCC patients compared with hepatitis patients and healthy controls, this highlighted that IL36 was probably mainly from the CD4^+^ T cells of PBMC. Growing evidence from Th cell-mediated disorders, such as inflammatory bowel diseases suggests IL-36γ binds to receptors on CD4+ T cells, potently inhibiting Foxp3-expressing induced regulatory T cell (Treg) development, while IL36 could promote Th9 cells differentiation via a IL-2-STAT5 and IL-4-STAT6-dependent pathway 27. Tregs are important immunosuppressive cells 28, and Th9 cells represent a unique subset of CD4+ T Cells endowed with the ability to eradicate advanced tumors 29, these could become potential mechanisms for IL36 in CD4^+^ T cells to induce the antitumor effector, and may explain higher expression of IL36 in HCC with better prognosis. To our knowledge, this is the first report describing the relationship of IL36 expression and the prognosis HCC. But, it is still very necessary to carry out multi-center trials with various tumor markers to confirm our conclusion, and the molecular mechanism underlying IL-36 and HCC still needs to be illuminated. In conclusion, our research suggested that the expression of IL-36 was significantly higher in HCC tissues compared with paired normal tissues, and low expression of IL-36 could suggest poor prognosis of HCC patients, rendering it a potential prognostic factor, biological marker of HCC, although this still needs to be validated in multicenter clinical study with larger scale and more samples.

## Figures and Tables

**Table 1:** Different expression level of IL36 in HCC tissues and peritumor tissues

		Expression of IL36 protein		
Tissue	Case NO	Positive	Negative	Positive rate
HCC	150	42	108	28%
Normal	150	127	23	85%

**Table 2:** Correlation between IL36 expression and clinical pathological features of HCC patients

	IL36 protein expression		χ^2^	*P* value
	positive	negative		
**Gender**			0.0029	0.957
Male	16	48		
Female	20	66		
**Age (years)**			0.3477	0.555
≤ 55	21	58		
>55	15	56		
**Liver cirrhosis**			5.6328	0.0176
Yes	14	72		
No	22	42		
**TNM classification**			1.4914	0.222
I-II	20	48		
III-IV	16	66		
**Tumor size (cm)**			1.73433	0.187859
≤4.5	20	48		
>4.5	16	66		
**Metastasis**			7.8028	0.005
Yes	40	51		
No	12	47		
**AFP (ug/L)**			5.6328	0.018
<400	23	45		
≥400	13	69		
**HBV infection**			4.771	0.029
Yes	23	47		
No	13	67		
**HCV infection**			4.303	0.038
Yes	16	28		
No	20	86		
**Child-pugh classification**			1.0705	0.3
A	20	50		
B	16	64		
**Edmondson grade**			3.3458	0.067
III	24	54		
IV	12	60		
**Surgical intervention**			7.21	0.007
Radical resection	10	63		
Palliative resection	26	51		
**Post-operative TACE**			5.633	0.018
Yes	22	42		
No	14	72		
**Total**	**36**	**114**		

**Table 3:** Univariate and multivariate analyses of clinical parameters and overall prognosis for HCC patients

Multivariate analysis of clinical parameters			
	HR (hazard ratio)	95% CI	*P*
**Age**	0.511	0.337-0.777	0.002
≤55 year vs >55 year			
**IL36**	0.432	0.271-0.688	0
Positive vs Negative			
AFP	1.722	1.149-2.679	0.008
≤400 ng/ml vs >400 ng/ml			
**Univariate analysis of clinical parameters**			
	**HR (hazard ratio)**	**95%CI**	***P***
**Gender**	0.691	0.483-0.988	0.043
Male vs Female			
**Age (years)**	0.661	0.458-0.954	0.027
≤55 vs >55			
**Tumor size (cm)**	1.026	0.717-0.501	0.069
≤4.5 vs >4.5			
**Liver cirrhosis**	1.541	1.076-2.207	0.018
Yes vs No			
**TNM stage**	0.717	0.501-1.026	0.069
I-II vs III-IV			
**Metastasis**	1.331	0.928-1.909	0.121
Yes vs No			
**HBV**	1.194	0.837-1.703	0.329
Yes vs No			
**HCV**	1.304	0.896-1.898	0.165
Yes vs No			
**Child-pugh class**	0.693	0.484-0.993	0.046
A class vs B class			
**Edmondson class**	0.814	0.568-1.167	0.263
III vs IV			
**Surgery**	0.538	0.374-0.774	0.001
Radical resection vs palliative resection			
**Post-operative TACE**	1.222	0.856-1.743	0.27
Yes vs No			
**IL36**	0.393	0.247-0.626	0
Positive vs Negative			

## Abbreviations

HCC: Hepatocellular Carcinoma
IHC: Immunohistochemistry
ELISA: Enzyme-linked Immunosorbent Assay
FCM: flow cytometry
GHE: Global Health Estimates
AFP: Alpa-feta protein
BCLC staging: Barcelona-Clinic Liver Cancer Group staging
TACE: transcatheter arterial chemoembolization
